# Impact of Positive Emotion Regulation Training on Negative Symptoms and Social Functioning in Schizophrenia: A Field Test

**DOI:** 10.3389/fpsyt.2019.00532

**Published:** 2019-07-26

**Authors:** Jérôme Favrod, Alexandra Nguyen, Anne-Marie Tronche, Olivier Blanc, Julien Dubreucq, Isabelle Chereau-Boudet, Delphine Capdevielle, Pierre Michel Llorca

**Affiliations:** ^1^La Source, School of Nursing Sciences, HES-SO University of Applied Sciences and Arts of Western Switzerland, Lausanne, Switzerland; ^2^CHU Clermont Ferrand, Service de Psychiatrie B, Université Clermont Auvergne, Clermont-Ferrand, France; ^3^Fondation FondaMental, Créteil, France; ^4^Centre Référent de Réhabilitation Psychosociale et de Remédiation Cognitive, CH Alpes Isère, Grenoble, France; ^5^Département de Psychiatrie Adulte, Centre Hospitalier Universitaire de Montpellier, Montpellier, France

**Keywords:** anhedonia, apathy, schizophrenia, negative symptoms, social functioning, positive psychology, emotion regulation, field test

## Abstract

**Background:** The poor efficacy of drug or psychological treatments on the primary negative symptoms of schizophrenia has led to the development of new interventions. The Positive Emotions Program for Schizophrenia (PEPS) is an emotion regulation strategy training that aims to intensify positive emotions and develop positive performance beliefs. A randomized controlled trial showed that PEPS is effective in reducing the composite score of the reduction of experience syndrome (anhedonia and apathy). The present study is designed to evaluate its feasibility in natural conditions to measure external validity of PEPS.

**Materials and Methods:** Twenty-one participants recruited through the French national network of expert centers followed eight sessions of PEPS and were assessed pre- and posttest with the Scale for Assessment of Negative Symptoms (SANS) and the Personal and Social Performance (PSP). The scales of the SANS were divided into a composite score of the reduction of the ability to experience and a composite score of the reduction of expression.

**Results:** All participants followed the 8 sessions of PEPS, and both composite scores were significantly and clinically improved at posttest. Social functioning assessed with the PSP was also improved.

**Conclusions:** This field test shows that participation in PEPS is accompanied by a reduction of negative symptoms and an improvement of social functioning. Both negative syndromes, reduction of expression and reduction of experience, are improved. Participants are younger than those in previous studies, which may explain this unexpected result. However, this calls for a controlled study with younger participants.

## Background

Negative symptoms in schizophrenia are characterized by marked reductions in reward-seeking behavior despite a seemingly intact capacity to experience pleasure (1). This reduction of behavior, which can include speech, nonverbal, and social behavior, hampers daily life functioning and interferes with the recovery process (2–5). Behavioral studies using reinforcement paradigms or effort tasks indicate that motivational impairments in schizophrenia might be associated with abnormalities in estimating the “cost” of effortful behavior (6). Reduction of reward-seeking behaviors is strongly correlated with effort discounting of monetary rewards that require physical effort but is not related with reduction of verbal or nonverbal expression (7). Patients with motivational deficits can fail to represent the magnitude of a reward when the difference between the probability and the magnitude of the reward gradually increase, suggesting an inability to combine information from different reward modalities (8).

Neuroimaging studies investigating the association between reward anticipation and motivational deficits indicate that hypoactivation of the dorsal caudate activity during reward anticipation is significantly associated with avolition, but not with anhedonia, in the patient group (9). Reduced responses to positive feedback in the dorsolateral prefrontal cortex and caudate have been observed among those patients higher in anhedonia/avolition (10). Ventral striatal activation during reward anticipation showed a strong negative correlation with apathy, but no significant correlation with the diminished expression dimension was observed (11). Behavioral and neuroimaging studies suggest that reductions in reward-seeking behavior are associated with prefrontal and ventral striatal areas implicated in planning complex cognitive behavior, decision making, and motivation.

Recent literature has distinguished negative symptoms indicating a reduction of the capacity to experience (apathy, anhedonia) from those that convey a reduction of expression (emotional blunting, alogia) (7, 12–14). The apathy–anhedonia syndrome tends to be associated with a poorer prognosis than the symptoms related to diminished expression, suggesting that it is the more severe facet of the psychopathology (14). This syndrome is also related to the duration of untreated psychosis, family history of schizophrenia, and the patient’s work status in first-episode schizophrenia (15). Behavioral and neuroimaging studies tend to show the underpinnings of these clinical differences (7, 11). Reduced frontostriatal functional connectivity associated with negative symptoms might provide a supplementary hypothesis to study the neurobiological mechanisms of negative symptoms (16).

As the efficacy of drug-based treatments and psychological interventions on primary negative symptoms remains limited (17–19), there is thus a clear clinical need for developing treatments for primary negative symptoms. Primary negative symptoms comprise the core features intrinsic to schizophrenia. Secondary negative symptoms are transient and attributable to the effects of factors such as unrelieved positive symptoms, depression, extrapyramidal side effects of antipsychotic drugs, or social isolation (20, 21).

The Positive Emotions Program for Schizophrenia (PEPS) is an emotion regulation strategy training that aims to increase the frequency, intensity, and duration of positive emotional experiences and to develop positive performance beliefs. These emotion regulation strategies include anticipating or remembering enjoyment, expressing emotions *via* nonverbal behaviors, directing controlled attention towards positive experiences when they occur, and sharing positive experiences with others. Participants are also trained to manage defeatist performance beliefs into positive ones. PEPS is an intervention consisting of 8 one-hour sessions applied to groups of 5–10 participants. The aim of the program is to increase cognitive control of positive emotions, including the anticipation and maintenance of those emotions. The program uses visual and audio materials as part of a PowerPoint presentation of slides projected onto a screen. The program was first tested in a pilot study (22) and in an independent study in Egypt with a culturally modified version (23). A recent randomized clinical trial showed that PEPS is effective in reducing diminished experience syndrome in patients with primary negative symptoms (24). However, when looking at anhedonia and apathy independently, PEPS is mainly effective in reducing anhedonia. This result is particularly interesting since the intervention focuses on maximizing positive emotion experiences. Patients could learn positive emotion regulation skills despite potential cognitive impairments in working memory and long-term memory. Following these results, the present study was designed to explore the feasibility of PEPS in natural conditions. In applied disciplines such as clinical psychiatry, the external validity must be emphasized to know about the efficacy of an intervention not only in experimental conditions but also in less controlled and more real-life situations. Clinical intervention should be effective and feasible under both conditions in order to be successfully used in everyday clinical practice (25). According to a pilot study (22), it was hypothesized that participating in 8 sessions of PEPS would improve the capacity to experience (anhedonia, apathy) of participants with schizophrenia.

## Materials and Methods

### Field Test Design

This open field test is a pre–posttest design used to assess the feasibility and replicability of PEPS in natural conditions.

### Subjects

Participants were recruited from the French national network of expert centers (26) in the Service de psychiatrie adulte B, Centre Hospitalier Universitaire de Clermont Ferrand, the Centre Référent de Réhabilitation (C3R), Grenoble, and the Département de psychiatrie adulte, Centre Hospitalier Universitaire de Montpellier. These services are part of the FondaMental Advanced Centers of Expertise-Schizophrenia (27). To be included, participants had to fulfill the *Diagnostic and Statistical Manual of Mental of Mental Disorders, Fourth Edition* (*DSM-IV*) criteria for a diagnosis of schizophrenia or a schizoaffective disorder, have a score greater than 1 on the Scale for Assessment of Negative Symptoms (SANS) apathy global score or the SANS anhedonia global score, and be interested in participating in PEPS. Participants with active psychotic relapse were not invited to participate. Substance use disorders were not an exclusion criterion.

All patients received a complete clinical psychiatric evaluation based on the Structured Clinical Interview for *DSM-IV* (28). General practitioners or psychiatrists can refer their patient in expert centers for detailed assessment and suggestions for therapeutic interventions. The goal is to supply a “personalized medicine” approach to the general population of schizophrenia patients. This comprehensive assessment aims to deliver advice to increase adherence, prevent relapse, and restore social functioning to improve long-term prognosis. All patients who meet diagnostic criteria for any schizophrenia or schizoaffective disorder could benefit from this examination. A web-based application, e-schizo, was developed by the FondaMental Foundation to collate assessment data for clinical monitoring and research purposes. The ethical committee and the committee in charge of the safety of computerized databases [Commission Nationale de l’Informatique et des Libertés (CNIL)] gave its approval and carefully regulated the access to the system. The XML format was used to transfer data from e-schizo into an anonymous national database (26). The study was approved by the relevant ethical review board [Comité de Protection des Personnes (CPP) Ile de France IX] on January 18, 2010. All participants gave their written informed consent.

## Intervention

### Positive Emotions Program for Schizophrenia (PEPS)

PEPS is an intervention designed to reduce anhedonia and apathy. The program teaches skills to help overcome defeatist thinking and to increase the anticipation and maintenance of positive emotions. Jérôme Favrod and Alexandra Nguyen conceived the intervention. The development of the intervention and the program description had been published previously (1). The French version of PEPS is freely downloadable at www.seretablir.net/peps/. PEPS comprises 8 one-hour group sessions, given once a week for eight weeks, administered using visual and audio materials and presented as PowerPoint presentation slides projected onto a screen. The skills taught include changing defeatist performance beliefs into more positive expectations, expressing emotions by increasing behavioral expression, savoring pleasant experiences, sharing pleasant experiences with others, and anticipating pleasant moments in the future. To familiarize the participant with the training materials, each session starts with a brief relaxation exercise using slowed breathing and mental focalization on savoring a pleasant experience

The intervention uses a collaborative, egalitarian approach. Group facilitators take part in sessions just as the participants do by performing the exercises, sharing their experiences, and conducting the given tasks. This style of animation allows group leaders to act as models and promote group members’ involvement and active participation (29). Group leaders can show how to disclose a positive experience in paying attention to details and describing what is pleasant in the experience. They can describe their own difficulties and how they overcome them. The pedagogical concept underpinning PEPS was designed according to Kolb and Kolb’s model (30) of experiential learning. This model sees the learning process as the transformation of an experience into personal knowledge. The sequential organization of the learning activity starts with the learner going through an experience (the concrete experience phase). Next, group leaders invite the learner to describe this experience to give it meaning (the reflective observation phase). Distancing oneself from the experience broadens the learner’s understanding, generalizing and developing concepts through more abstract thought (the abstract conceptualization phase). The learner then starts an experimental approach to confirm the newly acquired knowledge through reality tests (the active experimentation phase). The learning activities for each skill taught in PEPS always go ahead through these four steps. Group facilitators receive a day’s training before leading the intervention. Training was insured by AN and JF during a monthly clinical meeting of the schizophrenia expert group of the FondaMental Foundation. Group leaders were clinicians working at different sites (psychiatrists, psychologists, neuropsychologists, and nurses).

### Outcomes

#### Main Outcome

##### Measures

The following data and scales were used at T0 (pretest) and T1 (posttest) as part of standardized interviews with an independent evaluator trained in their administration. The average time needed to complete the scales with the participants was one hour.

The *Scale for Assessment of Negative Symptoms* (SANS) (31) measures schizophrenia’s deficit symptoms within the framework of schizophrenic disorders. It includes 25 items, scored from 0 to 5 (0 = none, 1 = questionable, 2 = mild, 3 = moderate, 4 = marked, 5 = severe). A definition of each item, including examples, facilitates a better understanding of the scale’s content. The rating system is ordinal, from 0 (absent) to 5 (severe). The 25 items are grouped into five subscales: (1) withdrawal or emotional poverty; (2) alogia (lack of speech); (3) avolition and apathy (lack of energy and lack of initiative); (4) anhedonia and social withdrawal (loss of interests); and (5) attention. A score is given to each component. The results can be expressed in different forms: an overall score (0 to 125), the subscores of each component, the sum of all subscores, or the sum of the five items of the overall evaluation. The scale was translated into French with acceptable validity (32, 33). A reduction of expression composite score was created in summing the items of the affective flattening subscale and dividing by 7 and in summing the items of the alogia subscale and dividing by 4. A reduction of the capacity to experience composite score was created similarly in summing the items of the avolition and apathy subscales and dividing by 3 and of the items of the anhedonia and social withdrawal subscale of the SANS and dividing by 4 (24).

The *Personal and Social Performance scale* (PSP) is a clinician-reported measure of the severity of personal and social dysfunction in a stabilized outpatient population with schizophrenia (34). Different areas of personal and social dysfunction are combined into four areas: personal and social relationships, socially useful activities, self-care, and disturbing and aggressive behaviors. Each of the four domains of behavior is assessed separately using a 6-point severity scale with the following categories: “absent,” “mild,” “manifest,” “marked,” “severe,” and “very severe.” The PSP delivers a single, overall rating score ranging from 1 to 100, where higher scores represent better personal and social functioning. Scores between 71 and 100 reflect no dysfunction or mild difficulties. Scores between 51 and 70 reflect dysfunction or difficulties ranging from manifest in one or more domains to marked in one domain. Scores between 1 and 50 reflect marked to very severe difficulties in two or more domains, whereas scores ≤30 demonstrate such poor social function as to require intensive support or supervision (34). The French validation of the PSP shows that the scale is reliable and valid for assessing the social functioning of patients with schizophrenia during the course of treatment as well as during acute phases (35).

The *Calgary Depression Scale for Schizophrenia* (CDSS) (36) includes nine items: depression, hopelessness, self-depreciation, guilty ideas of reference, pathological guilt, morning depression, early wakening, suicide, and observed depression with a global score range of 0–27 points. This scale has been validated in French (37). Participants with a score greater than 6 are considered depressed.

The *Temporal Experience of Pleasure Scale* (TEPS) contains 18 items included in two subscales: anticipatory pleasure (10 items) and consummatory pleasure (8 items) (38, 39). Items targeting anticipatory pleasure reflect the pleasure felt when anticipating a positive or pleasant stimulus. Items measuring consummatory pleasure refer to the direct and immediate pleasure experienced upon exposure to a stimulus. Items can be general or specific. Responses to items fall on a six-point Likert scale from 1 (“very false for me”) to 6 (“very true for me”). Both the total anticipatory and consummatory scores of TEPS were used. This scale is validated in French (40).

The *Savoring Beliefs Inventory* (SBI) is a self-assessment questionnaire composed of 24 items divided into three temporal orientations, past, present, and future, each represented by 8 items (41). Half of the items are positively formulated, while the other half are negatively framed. Each item is rated on a 7-point Likert scale ranging from “strongly disagree” to “strongly agree.” The total score of the SBI is calculated by subtracting the sum of the score of the negatively framed items from the sum the score of the positively phrased items. The SBI comprises three subscales: the anticipating pleasure subscale measures savoring a future positive event beforehand, the present-moment pleasure subscale measures enjoying positive events when they occur, and the reminiscing pleasure subscale measures recalling past positive events after they have occurred. The SBI has been translated into French and validated. The French version of the SBI is a valid scale to measure attitudes regarding the ability to savor positive experience, whether in anticipation or reminiscence or at the present moment (42).

Clinical team members from each center have monthly meetings to ensure good interrater reliability, to provide training in new therapeutic interventions, and to initiate new research studies.

### Statistical Analysis

All analyses were conducted using the IBM SPSS Statistics package version 22. Comparisons between paired *t*-tests were conducted to describe the differences between measures pre- and postintervention. Effect sizes were calculated using paired t-test statistics, using the formula reported by Borenstein and taking into account the dependence between data points (43).

## Results

Twenty-three participants were included, 7 in Montpellier, 10 in Grenoble, and six in Clermont-Ferrand expert centers. Participants were recruited through invitation by the team of the expert center. One participant wanted to participate but finally declined because of his time schedule, and another participant quit the study at the third session of PEPS saying that he did not want to participate anymore. The final sample was composed of 5 women and 16 men. The mean age was 33 years (SD 11.34). Eighteen fulfilled the criteria for *DSM-IV* schizophrenia, and 3 fulfilled the criteria for a schizoaffective disorder. Their mean duration of illness was 104 months (10 to 396). Four participants were in partial hospitalization, and the others were outpatients. The mean dose of neuroleptic medication in chlorpromazine equivalents (CPZ-EQ) was 586.9 mg (SD 410.8). The mean score of the CDSS at intake was 3.67 (SD 4.13). All participants were single except one, and only one participant had competitive employment. All participants followed the 8 sessions of PEPS, except the one who dropped out. **Table 1** shows the pre- and posttest results on the different scales used. The composite score of the reduction of the capacity to experience syndrome was significantly and clinically improved (t (20) = 3.22, p = 0.004, Cohen’s d = 0.70), as was the composite score of the reduction of expression syndrome (t(20) = 2.95, p = 0.008, Cohen’s d = 0.64). The PSP was also clinically and statistically improved. The self-reported scales did not show a statistically significant difference in the posttest, except for the SBI present-moment pleasure.

**Table 1:** Pre- and Posttest Results from the Different Scales.

	Pretest mean (SD)	Posttest mean (SD)	*t*	df	2-tailed p	Cohen’s d
**Primary outcomes**						
*SANS composite score of the reduction of the capacity to experience*	4.49 (1.74)	3.67 (1.61)	3.22	20	0.004	0.70
*SANS composite score of the reduction of expression*	3.50 (1.72)	3.07 (.174)	2.95	20	0.008	0.64
**Secondary outcomes**						
Personal and Social Performance scale	52.76 (10.94)	55.00 (12.07)	–2.80	20	0.01	0.61
*TEPS anticipatory*	43.53 (8.32)	45.47 (7.60)	–1.67	18	0.11	0.38
*TEPS consummatory*	35.37 (6.70)	36.37 (5.66)	–0.98	18	0.34	0.22
*SBI anticipating pleasure*	8.10 (8.01)	8.95 (7.35)	–0.43	18	0.67	0.10
*SBI present-moment pleasure*	0.50 (11.56)	6.16 (11.05)	–2.49	18	0.02	0.53
*SBI reminiscing pleasure*	5.68 (7.92)	7.68 (10.07)	–1.24	18	0.23	0.29

SANS, Scale for Assessment of Negative Symptoms; TEPS, Temporal Experience of Pleasure Scale; SBI, Savoring Beliefs Inventory—French version.Discussion.

## Discussion

This field test shows a very low attrition rate and an excellent participation rate indicating that PEPS is acceptable in natural conditions of treatment. With one day of training, the group leaders were able to conduct the program in three different sites, indicating a good feasibility of the intervention. Data indicate that participation in PEPS is accompanied by an improvement of negative symptoms and social functioning. The improvement from PEPS in the reduction of the capacity to experience syndrome already has been shown in previous studies (22, 24). This study indicates an effect on the reduction of expression syndrome that was only partially shown before. The correlation between the composite scores of expression and experience syndromes at pretest is not significant (Spearman rho = 0.29, p = 0.20), indicating some independence between the two syndromes in this study (see **Table 2**). The present study also suggests that PEPS may influence social functioning. These differences may be due to differences in population. The present sample is younger than previous samples. In the randomized controlled trial (RCT) (24), it has been shown that PEPS may not be as effective in older patients. It is possible that younger patients may benefit more from the intervention. This aspect deserves to be studied in a controlled study. Contrary to the RCT, the apathy–avolition scale of the SANS significantly improved (t(20) = 3.57, p = 0.002). It is possible that younger patients with a shorter duration of reduced experience syndrome are still better able to modulate behaviors to seek rewards than older patients. For example, a study with first-episode psychotic patients did not demonstrate overall reduction in effort expenditure but displayed reduced willingness to expend effort for high-value/high-probability reward as compared to controls (44). Longitudinal studies are required to clarify the trajectory of effort allocation and its potential utility in predicting amotivation and functional outcomes with different durations of illness. The severity levels in the apathy–anhedonia composite score between the present study and the randomized controlled study are quite similar, 4.5 in the present vs. 4.08, and may not explain the difference between the two studies.

**Table 2:** Correlations Between Variables at Pretest-Spearman rho (2-sided p).

	CPZ-EQ	Blunted affect	Alogia	Avolition	Anhedonia	Expression	Experience
CDSS	0.05 (0.82)	0.14 (0.54)	0.00 (0.98)	0.13 (0.59)	0.21 (0.36)	0.09 (0.69)	0.20 (0.38)
CPZ-EQ		0.30 (0.19)	0.08 (0.75)	0.08 (0.74)	0.00 (0.98)	0.13 (0.58	0.06 (080)
Blunted affect			0.71 (0.00)	0.25 (0.27)	0.48 (0.03)	0.87 (< 0.01)	0.45 (0.04)
Alogia				0.05 (0.83)	0.16 (0.49)	0.95 (< 0.01)	0.20 (0.39)
Avolition					0.37 (0.10)	0.12 (0.61)	0.78 (< 0.01)
Anhedonia						0.30 (0.19)	0.83 (< 0.01)
Expression							0.29 (0.20)

CDSS, Calgary Depression Scale for Schizophrenia; CPZ-EQ, chlorpromazine equivalents; blunted affect, SANS withdrawal or emotional poverty; alogia, SANS alogia (lack of speech); avolition, SANS avolition and apathy (lack of energy and lack of initiative); anhedonia, SANS anhedonia and social withdrawal (loss of interests); experience, SANS composite score of the reduction of the capacity to experience; expression, SANS composite score of the reduction of expression.

The present study does not control for secondary sources of negative symptoms except for depression. As in the controlled study, the sample scored low on depression. The participants were on a higher dose of antipsychotic medication as measured by the CPZ-EQ. However, *post hoc* analysis showed no correlation between affective flattening and the CPZ-EQ (Spearman rho = 0.30, 2-tailed p = 0.19) as well as for the other subscales of the SANS. **Table 2** shows correlations between CDSS, CPZ-EQ, composite scores, and subscores of the SANS. No unexpected significant correlation appears. There was no measure of the level of psychotic symptoms in the present study intake. However, in the expert centers, the patients referred during a psychotic relapse were postponed until they had reached at least partial remission to be able to participate in the assessment.

The self-report scales did not show improvement except for the SBI present-moment pleasure scale. In a previous study (24), the TEPS anticipatory and TEPS consummatory scales were improved at posttest, but this was not the case in the present study. The SBI present-moment pleasure scale was improved at the six-month follow-up, but not at posttest (24). It is possible that participants needed time after the end of the program to observe a change in their own abilities to experience pleasure. The SBI present-moment pleasure scale was improved in the present field test in a similar manner as in the pilot study (22). This finding suggests that patients can improve the present-moment experience but have difficulties recalling past pleasure experience and anticipating pleasure in the future. These deficits might have several reasons and could be due to a less distinct preference for positive over neutral stimuli (45) or a difficulty relying on experiential emotion in a hypothetical self-report format (46). It also can be linked to other more general deficits in cognition (47). In addition, there are several studies reporting partly intact in-the-moment hedonic experience (pleasure) in patients with schizophrenia, interestingly even better in those with more negative symptoms (48, 49). Finally, a case study indicates that training patients to anticipate pleasure requires a much longer training to obtain an improvement on self-report scales (50).

In the present study, only two participants declined to participate, one at inclusion and one during the intervention. The participation rate was excellent since participants completed all sessions.

This study presents several limitations. It is an uncontrolled field test aimed at assessing the generalizability in a natural environment of previous results of a controlled study. The absence of control for secondary sources of negative symptoms apart from depression limits the strength of the observed changes. The small sample size and the lack of a control group do not allow us to draw firm conclusions. The absence of data on substance abuse may be a limitation because it restrains comparisons with other studies.

Patients assessed in expert centers were referred by their general practitioner or psychiatrist and received a detailed evaluation report along with suggestions for therapeutic interventions; exclusion criteria for referral are minimal (26). Eighty-two percent of subjects referred to the expert centers received full clinical and neuropsychological assessments. Even if the participants were willing to participate in this study, they were drawn from a standard population of people with schizophrenia. In future studies, it would be useful to use assessment instruments such as the Clinical Assessment Interview for Negative Symptoms (CAINS) (51) or the Brief Negative Symptom Scale (BNSS) (52). However, the study was designed in 2015 and started in 2016, before these instruments became popular and available in French. The results of the present study call for a randomized controlled study with younger participants to evaluate the efficacy of PEPS earlier in the development of the illness.

In conclusion, this field test adds external validity to PEPS to improve the reduced capacity to experience syndrome in schizophrenia. It allows the generation of new useful hypotheses on the effect of PEPS on younger populations of patients and on social functioning.

## Data Availability

The dataset generated for this study is available from the corresponding author upon reasonable request and with permission of the Foundation FondaMental.

## Author Contributions

JF and AN in equal measure conceptualized PEPS, trained the group leaders, analyzed and interpreted the data, and drafted the first version of the manuscript. AN, JF, and PL conceptualized the study. PL, A-MT, and OB coordinated the study among the FondaMental Advanced Centers of Expertise-Schizophrenia. JD, IC-B, and DC acquired the data and facilitated the recruitment of patients and the intervention in their center. All authors gave a substantial contribution to the analysis and interpretation of data and critically revised the article for important intellectual content. All the authors approved the final version for publication. All the authors agree to be accountable for all aspects of the work by ensuring that any questions related to its accuracy are answered.

## Funding

This work was supported by the FondaMental Foundation and the Swiss National Science Foundation, grant number 105319_163355.

## Conflict of Interest Statement

The authors declare that the research was conducted in the absence of any commercial or financial relationships that could be construed as a potential conflict of interest.
